# Acute Toxoplasmosis in Two Immunocompetent Colombian Soldiers after Ingestion of Undercooked Squirrel Meat

**DOI:** 10.4269/ajtmh.24-0659

**Published:** 2025-01-07

**Authors:** Daniel Celis-Giraldo, Leidy J. Medina-Lozano, Álvaro A. Faccini-Martínez

**Affiliations:** ^1^ Facultad de Medicina, Universidad Militar Nueva Granada, Bogotá, Colombia;; ^2^ Servicio de Medicina Interna, Hospital Militar Central, Bogotá, Colombia;; ^3^ Group of Molecular Parasitology (GEPAMOL), Center for Biomedical Research, Universidad del Quindío, Armenia, Colombia;; ^4^ Servicio de Infectología, Hospital Militar Central, Bogotá, Colombia

**Figure 1.**
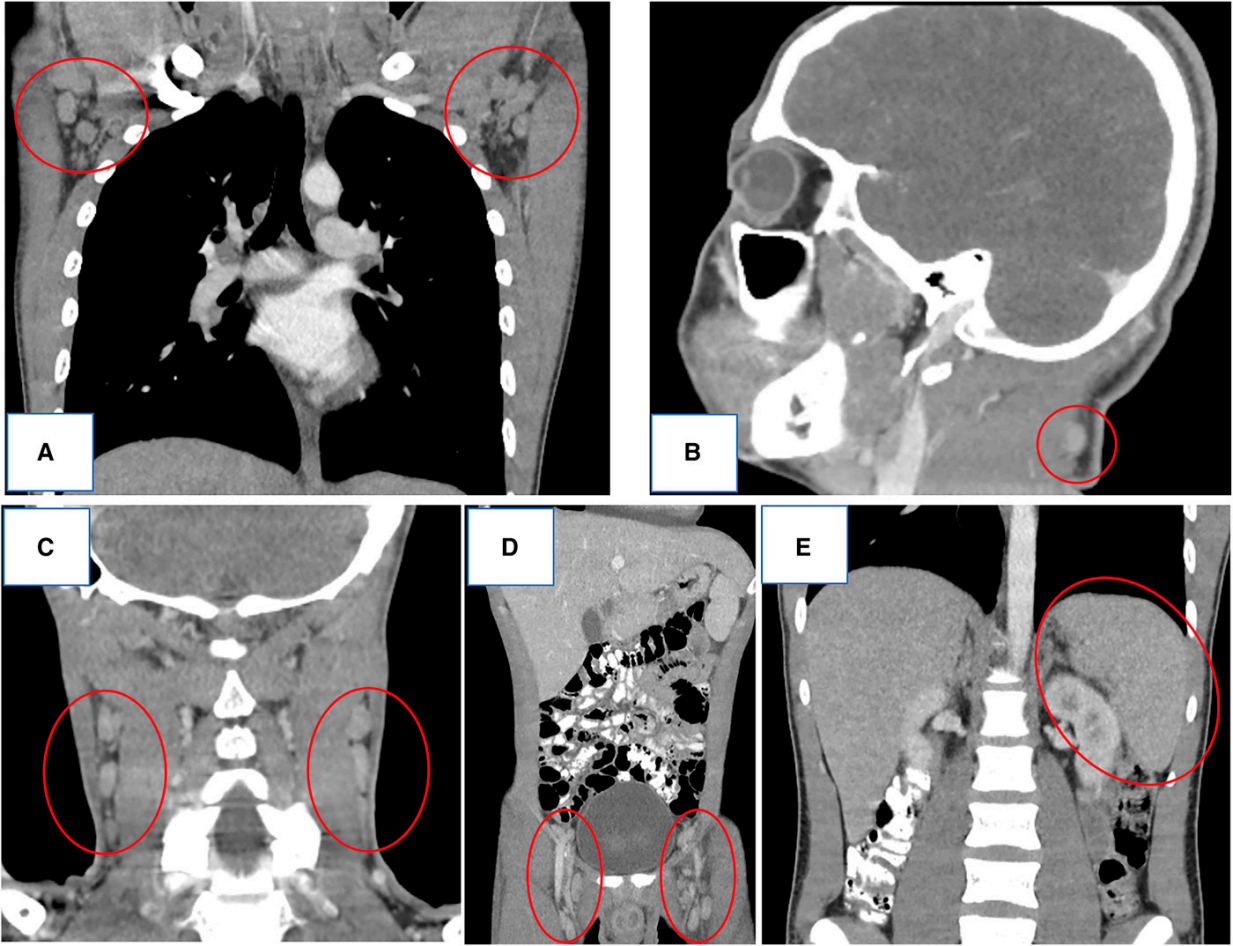
High-resolution tomography of the thorax and head (coronal and sagittal planes, respectively) showing (**A**) bilateral axillary lymphadenopathy and (**B**) an enlarged cervical lymph node in the first patient and high-resolution tomography of the head and abdomen (coronal planes) showing (**C**) bilateral cervical lymphadenopathy, (**D**) bilateral inguinal lymphadenopathy, and (**E**) splenomegaly (18 cm) in the second patient.

On December 5 and 15, 2023, two young, previously healthy soldiers stationed in the jungle of Vichada department (Amazon-Orinoco Colombian region) were transferred to our institution, Hospital Militar Central, for prolonged febrile illness. The first patient had 12 days of fever, generalized lymphadenopathy, asthenia, night sweats, and retinochoroiditis of the right eye with a whitish feathery edged lesion of 0.5 in diameter disk (dd) with perilesional vasculitis. The second patient had 22 days of fever, generalized lymphadenopathy, weight loss, diarrhea, and retinochoroiditis of the left eye with a whitish lesion on the upper temporal arcuate with undefined borders of 0.5 in dd. The patients reported eating undercooked squirrel meat and drinking untreated water during jungle patrols. In both patients, IgM and IgG antibodies to *Toxoplasma gondii* were positive, and there were low IgG avidity indices. Serological results were negative for acute HIV, Chagas, syphilis, Cytomegalovirus and Epstein-Barr virus infections. Computed tomography revealed generalized lymphadenopathy and splenomegaly in both patients (Figure 1). They received treatment with trimethoprim/sulfamethoxazole for 4 weeks and exhibited significant clinical improvement.

In a recent systematic review that described acute toxoplasmosis in immunocompetent hosts, classic manifestations included fever (86%), lymphadenopathy (74%), malaise (68%), and ocular findings (34%). The most common risk factors were eating undercooked meat (47%) and drinking untreated water (37%), similar to our cases.^1^ Outbreaks among Colombian military personnel have been reported.^2^ Toxoplasmosis in South America exhibits different clinical behavior, and treatment should be initiated in the context of acute infection.^3^
